# Yeast Complementation Assays Demonstrating the Importance of the Affinity Tag Position in Membrane Protein Purification, as Exemplified by *Hp*UreI, the pH‐Gated Urea Channel of *Helicobacter pylori*

**DOI:** 10.1002/smsc.202400571

**Published:** 2025-01-27

**Authors:** Anna Stoib, Sahar Shojaei, Christine Siligan, Andreas Horner

**Affiliations:** ^1^ Institute of Biophysics Johannes Kepler University Linz Gruberstr. 40 4020 Linz Austria

**Keywords:** affinity tag positions, *Helicobacter pylori*, *Hp* UreI, pH‐gated urea channels, yeast complementation assays

## Abstract

Affinity tags are a crucial component in protein purification. Despite several indications that they can influence protein structure and function, this influence is often unknown or disregarded. This unnecessarily introduces ambiguity in the interpretation of in vitro data. To illustrate that, urea and ammonia yeast complementation assays are used as a screening tool to assess functional differences in various affinity tag positions, compared to the WT protein, using *Hp*UreI, an acid‐gated urea channel of *Helicobacter pylori*. Yeast complementation assays test the pH‐dependent functionality of exogenous proteins expressed in deletion strains by observing growth. If the exogenous protein is able to replace the function of the deleted endogenous protein, yeast cells can demonstrate growth under specific assay conditions. The overall tag position and even a minor amount of residual N‐ or C‐terminal amino acids following tag cleavage exert a solute‐specific influence on *Hp*UreI functionality, suggesting a complex solute selectivity mechanism and underscores the necessity for in vivo characterization. This cost‐effective yeast complementation assay can be adapted to test a broad range of solutes. It can be used as a preliminary screening tool for affinity tag positions or protein mutations before quantitative in vitro protein characterization.

## Graphical abstract

Affinity tags help to purify proteins, but can interfere with protein function or structure. Yeast complementation assays to study *Helicobacter pylori's* UreI protein are used. Results indicate that the tag position and residual amino acids resulting from tag cleavage have a significant influence on protein permeability to urea and ammonia. This straightforward and cost‐effective method enables the testing of various solutes and tag variants prior to in vitro characterization.

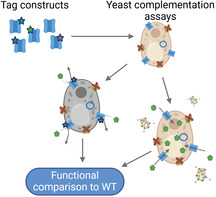

## 1 Introduction

Affinity chromatography is the predominant method for the purification of recombinant proteins. The most common method to achieve high yields of pure protein for in vitro characterization uses immobilized metal ion affinity chromatography in conjunction with a poly His‐tag.^[^ 1, 2, 3, 4 It is usually assumed that the relatively small tag of 2.5 kDa has a minimal impact on protein structure, stability, and function.^[^ ^5^, ^6^ ^]^ However, several publications have already demonstrated that the expression,^[^ ^7^, ^8^ ^]^ permeability,^[^ ^9^ ^]^ activity,^[^ ^7^, ^10^, ^11^, ^12^, ^13^, ^14^, ^15^ ^]^ oligomeric assembly,^[^ ^8^, ^16^ ^]^ crystal formation,^[^ ^17^ ^]^ structure,^[^ ^10^, ^13^, ^14^ ^]^ and protein stability^[^ ^10^ ^]^ are all adversely affected. Consequently, the properties of a protein may be influenced not only by the choice of tag but also by the position, length, and linker sequence.^[^ ^6^, ^14^, ^16^, ^18^, ^19^ ^]^ It would be erroneous to assume that these negative effects are universal to all proteins. However, the aforementioned studies demonstrate that any potential impact of protein modification must be considered and, where possible, excluded through experimental means. While the majority of protein studies do not consider the potential impact of the affinity tag, some studies have compared the tagged protein version with the poly His‐tag cleaved protein.^[^ ^6^, ^12^, ^13^, ^15^ ^]^ This strategy assesses the impact of the affinity tag but does not address the potential influence of the linker and residual amino acids following cleavage. To determine the native‐like functionality of the protein variant, a comparison to the untagged wild‐type (WT) protein is imperative.^[^ ^18^ ^]^

A variety of techniques can be employed in order to test the native‐like functionality of proteins. The production of pure, untagged WT proteins with a high yield is rare. One example employs the thermal precipitation of proteins, followed by ammonium sulfate precipitation.^[^ ^10^, ^11^ ^]^ However, this technique is only applicable to thermostable proteins. An additional avenue for investigation is the assessment of enzyme activity in crude cell lysates.^[^ ^7^ ^]^ This approach entails the monitoring of a specific reaction catalyzed by the overexpressed protein. Of broader applicability are functionality tests conducted in vivo, with proteins expressed in a host system. One possibility is to express the various tag constructs in *Xenopus laevis* oocytes.^[^ ^20^, ^21^, ^22^ ^]^ In this approach, mRNA is injected into the oocytes, which then express the protein of interest. The functional readouts include volume changes,^[^ ^23^, ^24^, ^25^ ^]^ current measurements,^[^ ^26^, ^27^, ^28^ ^]^ and radioactive uptake.^[^ ^20^, ^21^, ^22^, ^26^, ^27^ ^]^ It is important to note that the estimation of water permeabilities from volume changes, after application of hypo‐osmotic buffers to oocytes, is a topic of debate in the scientific community. This is due to the potential impact of unstirred layers and the interconnection between the membrane and the cytoskeleton, which can present an additional obstacle to volume changes.^[^ ^29^, ^30^, ^31^ ^]^ An additional option is to conduct complementation assays with yeast as a model organism. In this context, the growth or survival of yeast deletion strains is contingent upon the functionality of the overexpressed protein. Yeast complementation assays are a widely utilized method for the characterization of function,^[^ ^32^, ^33^, ^34^, ^35^, ^36^ ^]^ solute permeabilities,^[^ ^37^, ^38^, ^39^, ^40^, ^41^ ^]^ the linking of selectivity to amino acids,^[^ ^42^, ^43^ ^]^ and the examination of interactions with inhibitors or drugs.^[^ ^41^, ^44^, ^45^ ^]^ Consequently, complementation assays offer an extensive range of functional testing for a diverse array of proteins in a time‐ and cost‐effective manner.

*Saccharomyces cerevisiae*, also referred to as budding yeast, is a unicellular eukaryote. The majority of processes, including transcription, translation, and protein folding, are conserved, rendering it a cost‐effective alternative for complex mammalian cell culture.^[^ ^46^, ^47^ ^]^ With a duplication time of 90 min at standard conditions, *S. cerevisiae* exhibits a rapid growth rate, thereby enabling the investigation of several generations of a yeast population in a single experiment. Yeast can be cultivated in both liquid and solid cultures, and the requisite media ingredients are relatively inexpensive.^[^ ^48^ ^]^ In contrast to other model organisms, such as *Escherichia coli*, yeast is capable of expressing integral membrane proteins without experiencing toxicity issues.^[^ ^49^ ^]^ With respect to the lipid composition, the yeast membrane is more analogous to the plant cell wall than that of any other model organism.^[^ ^46^, ^50^ ^]^ The main phospholipids present are phosphatidylserine, phosphatidylinositol, phosphatidylethanolamine, and cholesterol, with a concentration of up to 15%.^[^ ^50^ ^]^ Following the sequencing of the yeast genome in 1996,^[^ ^51^ ^]^ a number of tools for genetic manipulation were developed, including shuttle vectors^[^ ^52^ ^]^ and methods of gene deletion by homologous recombination.^[^ ^53^ ^]^ Consequently, the majority of genes have been linked with their respective functions. Moreover, extensive libraries comprising deletion strains are readily available, and functional assays can be rapidly established.^[^ ^47^, ^54^ ^]^

**Figure 1:**
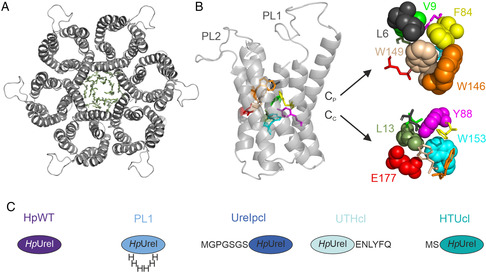
*Hp*UreI structure and tested tag constructs. A) *Hp*UreI assembles into a hexamer (PDBID: 3UX4 is shown from the periplasmic side). The central pore is occluded by lipids (green sticks). B) Each monomer constitutes a pore formed by six transmembrane helices exhibiting a threefold repetition of two helix hairpin motifs. The C and N‐termini are located in the periplasm, resulting in the formation of two periplasmic loops (PL) between transmembrane helices 2 and 3 (PL1) and transmembrane helices 4 and 5 (PL2).^[^ ^21^ ^]^ Two layers of hydrophobic residues (colored) form the channel's selectivity filter. The monomer illustrates an AlphaFold model that includes the structurally unresolved PLs. C) Variants of *Hp*UreI with residual amino acids at both the N‐terminus (UreIpcl and HTUcl) and C‐terminus (UTHcl), resulting from the cleavage of the His‐tag, are compared in yeast complementation assays to the untagged WT *Hp*UreI function throughout the manuscript. Additionally, a construct featuring a His6 tag in PL1 is included in the comparison (PL1).

In this study, yeast complementation assays are employed to investigate the impact of the affinity tag position on the function of *Hp*UreI, a proton‐gated inner membrane urea channel of *Helicobacter pylori* (**Figure** **1**). The protein facilitates the transport of urea into the cytosol of the pathogen, where the urease enzyme then hydrolyzes urea to ammonia and carbon dioxide. It is hypothesized that the products serve to buffer the pH of the cytoplasmic and periplasmic space, thereby enabling the bacteria to not only survive but also flourish in conditions of extreme acidity. This characteristic enables *H. pylori* to establish colonization within the human stomach at an average pH range of 2.^[^ ^20^, ^21^, ^22^, ^55^, ^56^ ^]^ It is estimated that ≈50% of the global population is infected chronically with *H. pylori*. Furthermore, it can be concluded that the Gram‐negative bacterium is a primary etiological agent for gastric diseases, including gastric ulcer disease and stomach cancer.^[^ ^57^, ^58^ ^]^ The current therapeutic approach employs a combination of proton pump inhibitors and multiple antibiotics.^[^ ^59^, ^60^ ^]^ However, the efficacy of this treatment is diminished,^[^ ^59^, ^61^ ^]^ due to the increasing prevalence of antibiotic resistances. An alternative approach would be to prevent the survival of *H. pylori* in the acidic environment of the stomach by targeting the hexameric inner membrane channel UreI with a drug.^[^ ^20^ ^]^ At an acidic pH of 5.0, *Hp*UreI, with a pKa of 5.9,^[^ ^55^ ^]^ is in an open state, whereas at pH 7.0 it is in a closed state, indicating that the buffering capacity is only required in an acidic environment. At higher pH values, the closure of the channel prevents the cytosol from becoming overly basic due to the continuous hydrolysis of urea by urease.^[^ ^20^, ^21^, ^22^, ^55^, ^56^ ^]^ In addition to urea, *Hp*UreI exhibits water permeability in a pH‐dependent manner analogous to that observed for urea.^[^ ^55^ ^]^ Furthermore, it has been proposed that the products of urease hydrolysis, namely ammonia (NH_3_) and ammonium (NH_4_^+^), as well as carbon dioxide (CO_2_), may permeate through *Hp*UreI.^[^ ^56^ ^]^

The functionality of *Hp*UreI tag constructs (Figure 1C) is compared to that of the untagged WT *Hp*UreI in pH‐dependent urea and ammonia growth assays utilizing *S. cerevisiae*. The relative permeabilities of urea and ammonia, as well as the pH gating mechanism of *Hp*UreI, are examined within the physiologically relevant pH range of 4.0–7.0. Our findings highlight the importance of in vivo screening, as both the overall tag position and residual N‐ or C‐terminal amino acids following poly‐His‐tag cleavage have an impact on *Hp*UreI's solute‐specific gating characteristics.

## 2 Results

### 2.1 Urea Complementation Assay

**Figure 2:**
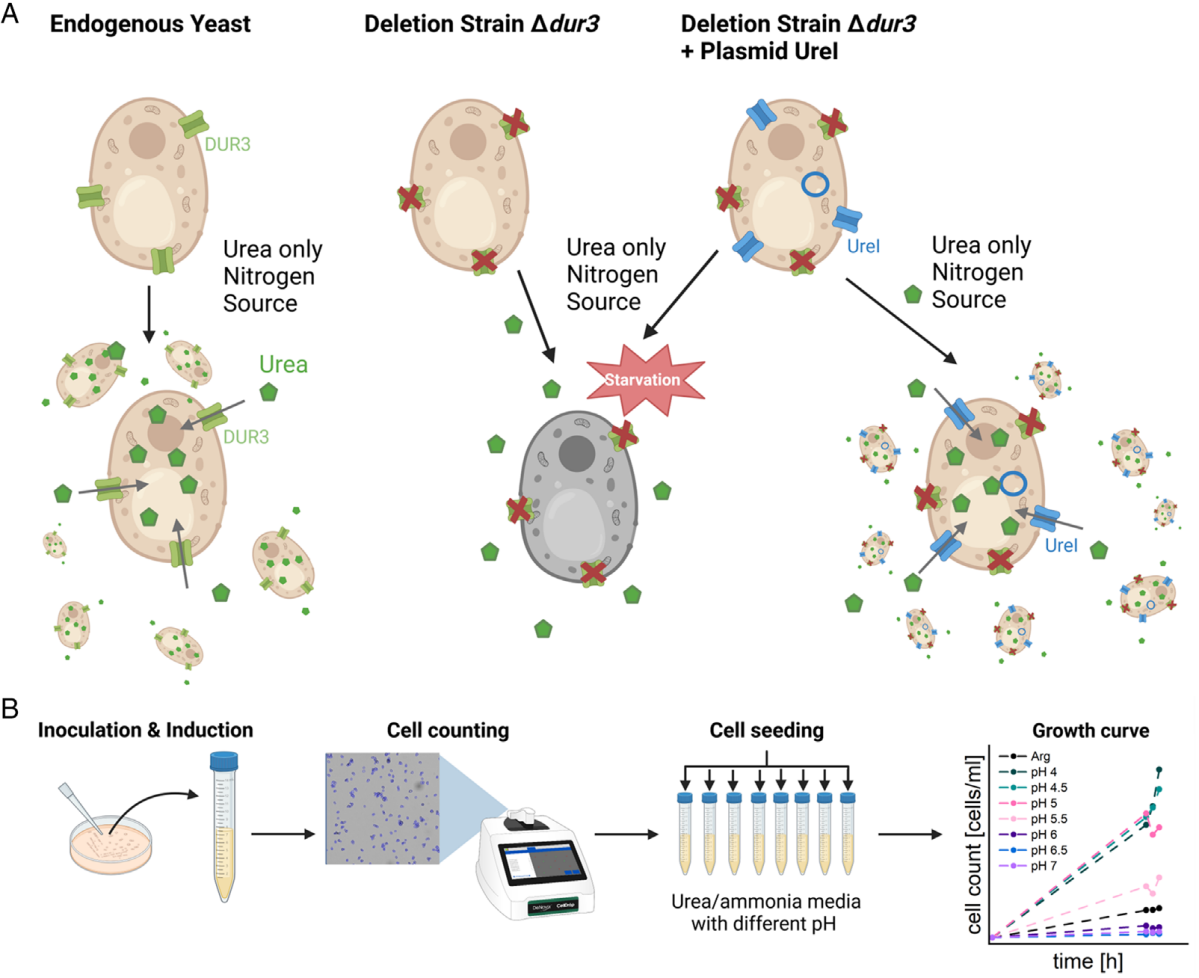
Urea complementation assays in *S. cerevisiae*. A) Assay principle: The endogenous yeast can survive in conditions with urea as the sole nitrogen source as the channel DUR3 permeates urea into the cell. In a Δ*dur3* deletion strain, urea can only enter the cell passively through the cell membrane, thereby limiting its availability as a nitrogen source. Subsequently, the cells enter a stage of starvation and show reduced growth rates. Hence, the functionality and urea permeability of a potential urea channel can be deduced from the growth of the transformed deletion strain. The greater the permeability of the urea channel, the better the growth of cells, as the channel complements the deletion of DUR3. B) Workflow: Single colonies of transformed constructs are inoculated, protein expression is induced by a change to galactose‐containing media for 24 h, and the same cell number is seeded into urea or ammonia media with a pH range of 4.0–7.0 and arginine media as control. Cell concentrations are measured 2 days after seeding.

To observe relative variations in permeability and gating between the different affinity tag constructs (Figure 1) cell growth is measured within a pH range of 4.0–7.0 in increments of 0.5 pH units. To conduct urea complementation assays, a yeast strain (Δ*dur3*) that is deficient in urea uptake is transformed with a plasmid that carries the tag constructs of *Hp*UreI. The transformed yeast cultures are cultivated in media containing urea as the sole nitrogen source, and cell growth is monitored. If the channel is permeable for urea, the yeast cells will obtain nitrogen and thus complement the deficient yeast strain and exhibit accelerated growth.^[^ ^33^, ^34^, ^36^, ^62^ ^]^ The principle of the urea complementation assay is illustrated in **Figure** **2**. To serve as a control for the inner urea metabolism, arginine is employed as a nitrogen source, as it is metabolized to urea following cell uptake^28^ (Figure S1, Supporting Information). Therefore, the relative urea permeabilities of *Hp*UreI variants that complement the deletion strain Δ*dur3* illustrate the growth advantage of cells that utilize urea as a nitrogen source. We found that the optimal incubation for measuring differences in urea permeabilities is 2 days after seeding. During this time, the culture remains in the exponential growth phase, allowing for the manifestation of growth pattern differences across several generations.^[^ ^63^ ^]^ Prolonged incubation results in a decline in cell count due to cell death (Figure S2, Supporting Information).

**Figure 3:**
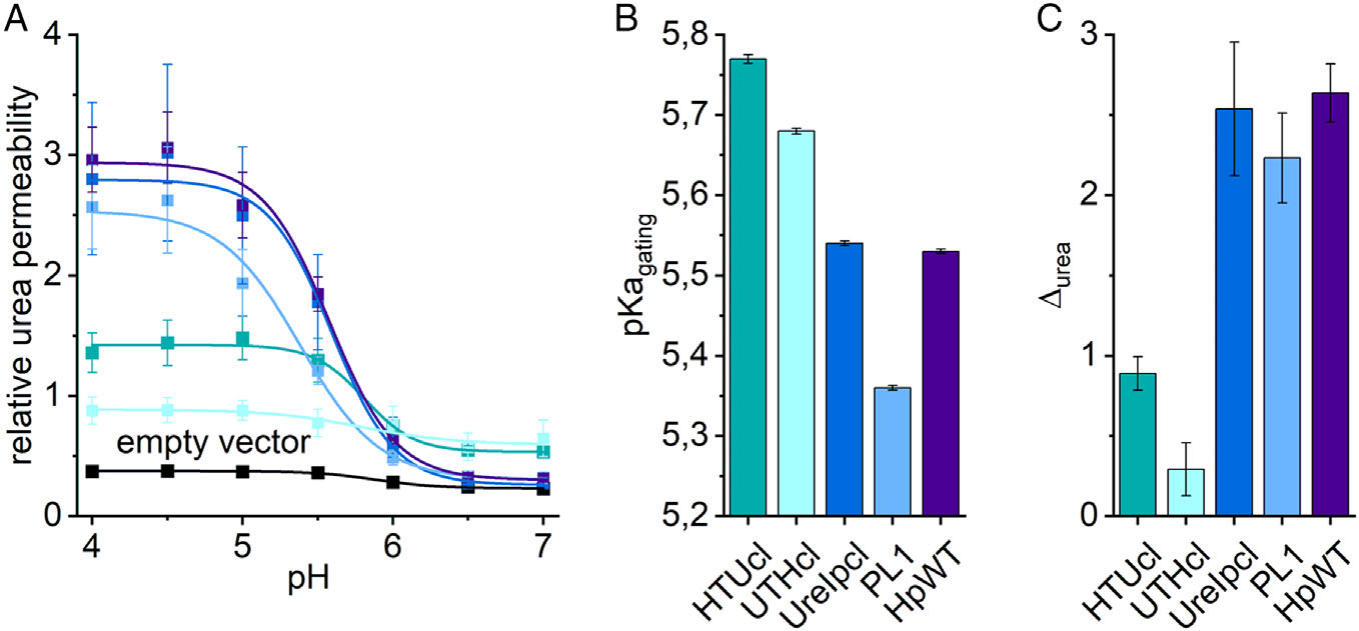
Relative urea permeabilities of the tag constructs compared to *Hp*UreI WT. A) The averages ± SEM (standard error of means) of the relative urea permeability of seven independent assays are presented for empty vector, WT *Hp*UreI, and tag constructs (N‐terminal HTUcl, UreIpcl; C‐terminal UTHcl and intraloop PL1). Data points were fitted with a Boltzmann function. B) Inflection points of the fits to the pH dependencies in (A), show the pKa values for channel opening and closing. C) The changes in relative urea permeabilities between pH 4.0 and 7.0, Δ_urea_, are shown.

The utilization of urea complementation assays has demonstrated that the untagged WT *Hp*UreI exhibits the noted pH‐dependent gating behavior for urea, as previously observed by others.^[^ ^20^, ^21^, ^55^ ^]^ Figure 2B illustrates exemplary growth curves at different pH conditions. *Hp*UreI displays permeability to urea at acidic pH and undergoes closure at neutral pH. At a pH of 5.53 ± 0.003 (**Figure** **3**A,B), approximately half of the channels are open, which is in close proximity to the pKa of 5.9, as was previously observed in oocytes.^[^ ^56^ ^]^ As a negative control, cells transformed with the empty vector backbone of pYES2 exhibited an overall low relative urea permeability (Figure S3, Supporting Information, Figure 3A). The introduction of the His‐tag into PL1, in a manner analogous to that employed for the structural^[^ ^20^, ^21^ ^]^ and functional studies,^[^ ^55^ ^]^ results in a reduction of *Hp*UreI's urea permeability and a shift in its pKa of opening/closing to 5.35 ± 0.003. This observation is consistent with the data presented by Strugatsky et al.^[^ ^21^ ^]^ It is noteworthy that residual amino acids of a TEV cleavage site at the N‐ and C‐termini have a profound impact on the functionality of *Hp*UreI. The pKa of opening/closing is shifted toward the neutral pH region (e.g., 5.77 ± 0.005 for HTUcl) and the change in relative urea permeability, Δ_urea_, between the open and the closed state of the channel is reduced to only 30 and 10% for HTUcl and UTHcl, respectively, relative to the WT protein. Furthermore, the channels are only partially closed at neutral pH (Figure 3). Notably, the insertion of four linker residues at the N‐terminus between UreI and the HRV cleavage site results in the restoration of the WT behavior with regard to relative urea permeability and the pKa value of opening/closing. These results highlight the importance of the tag position and residual amino acids at both termini and within PL1, but also illustrate their pivotal role in the pH‐gating mechanism of *Hp*UreI. To exclude the possibility that the reduced urea permeabilities of HTUcl and UTHcl at an acidic pH are caused by the protein expression level of the respective construct, we employed Western blot analysis utilizing an anti‐*Hp*UreI antibody. The analysis revealed small differences in expression levels between the individual constructs and assays; however, protein expression was consistently detectable. From these variations in combination with the reasonably small standard errors of means in Figure 3A it was not possible to identify any significant differences in protein expression levels, suggesting that the variations in the amino acid sequence of the constructs are responsible for the observed reduction of permeability at acidic pH (Figure S4, Supporting Information).

### 2.2 Ammonia Complementation Assay

**Figure 4:**
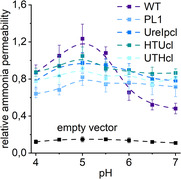
Relative ammonia permeabilities of the tag constructs compared to *Hp*UreI WT. Averages ± SEM of relative ammonia permeability of four independent assays for empty vector, WT UreI, and tag constructs (N‐terminal HTUcl, UreIpcl, C‐terminal UTHcl, and loop PL1). Spline lines help to guide the eye.

The primary function of *Hp*UreI is to facilitate passive urea permeability across the inner membrane in a pH‐dependent manner. Nevertheless, it has been demonstrated that *Hp*UreI is also capable of facilitating ammonia permeability.^[^ ^56^ ^]^ To ascertain the functional implications of our tag constructs with regard to potential ammonia permeability, we conducted ammonia complementation assays in a manner analogous to the urea complementation assays previously described. Ammonia complementation assays are conducted by transforming an ammonia uptake‐deficient yeast strain (Δ*mep1‐3*) with a plasmid carrying varying *Hp*UreI constructs. The transformed yeast cultures are cultivated in media containing ammonia as the sole nitrogen source and cell growth is monitored over time. If the respective channel is permeable to ammonia, the yeast cells will obtain nitrogen, thereby complementing the deficient yeast strain and exhibiting accelerated growth.^[^ ^35^, ^38^, ^42^ ^]^ The principle of the ammonia complementation assay is illustrated in Figure S5, Supporting Information. Once more, arginine is employed as a nitrogen source as a control for the yeast's internal nitrogen metabolism, given that ammonia is synthesized internally,^[^ ^34^ ^]^ when arginine is metabolized (Figure S1, Supporting Information). The WT UreI protein is able to complement the deletion strain Δ*mep1‐3*, as it enables the cells to grow under arginine and ammonia as the sole nitrogen source (Figure S6, Supporting Information). These measurements demonstrate that *Hp*UreI is indeed permeable to ammonia, exhibiting a pronounced pH dependence (pKa ≈ 5.7). As with urea, its permeability is increased in the acidic pH range, yet it remains partially open to ammonia at neutral pH (**Figure** **4**). Even at neutral pH, the relative ammonia permeability is significantly above the background permeability (black line). Furthermore, a decrease in relative ammonia permeability is observed at pH values below 5.0. This may also indicate a reduction in ammonia availability at more acidic pH conditions as evidenced by the clear trend observed across all tag constructs including the control (Figure 4). Notably, ammonia permeability is independent of pH for all tag constructs, which suggests that *Hp*UreI may possess a complex solute selectivity mechanism. Similar to the urea complementation assays, Western blot analysis demonstrates similar expression levels of *Hp*UreI in ammonia complementation assays for all constructs (Figure S7, Supporting Information).

## 3 Discussion

*Hp*UreI consists of six protomers comprising 195 amino acids each, which fold into six membrane‐spanning helices. Consequently, both the N‐ and C‐terminus, as well as loops PL1 and PL2, face the periplasm of the Gram‐negative pathogen.^[^ ^21^ ^]^ The protomer possesses an hourglass shape, narrowing in the middle of the membrane into a constriction zone formed by two layers of hydrophobic residues containing a glutamic acid at position 177 (Figure 1C). Two molecular gating mechanisms have been proposed in literature based on cryoEM structures of the channel in the open and closed states,^[^ ^20^ ^]^ as well as molecular dynamics simulations.^[^ ^64^ ^]^ *Hp*UreI structures of the PL1 construct suggest that His131 on PL2 triggers a sequential and cooperative movement of PL1, PL2, and the C‐terminus. It is hypothesized that these changes result in the displacement of the transmembrane helices, which ultimately leads to the reorientation of the channel‐lining amino acid residues.^[^ ^20^ ^]^ In contrast, molecular dynamics simulations of the open state have revealed a folding event of PL1 into the pore, suggesting pore obstruction as a potential gating mechanism of *Hp*UreI.^[^ ^64^ ^]^

### 3.1 Urea Complementation Assay

Our results do not allow us to rule out one or the other gating mechanism. However, they highlight the crucial role played by all periplasmic structural elements in the molecular gating mechanism. The positions of the tag and slight differences in the length of the linkers of the UreI constructs were found to influence the permeability and pH gating of urea uptake. In comparison to WT UreI, which exhibits maximum urea permeability at pH 4.5 and a closed state at pH 6.5 with a pKa of 5.53 ± 0.003, the presence of residual amino acids (UreI‐ENLYFQ) at the C‐terminus (UTHcl) was observed to strongly decrease urea permeability at acidic pH and prevent a fully closed state at neutral pH. This finding is consistent with the proposed importance of the C‐terminus in channel gating.^[^ ^22^ ^]^ Additionally, residual amino acids (MS‐UreI) at the N‐terminus (HTUcl) also prevent channel closure at neutral pH, yet allow for intermediate permeability at acidic conditions. The pKa value for HTUcl was found to be shifted to 5.77 ± 0.005. In accordance with literature,^[^ ^20^ ^]^ the PL1 construct bearing the His‐tag within the PL1 loop exhibits a reduced permeability and a lower pKa value of 5.35 ± 0.003. Interestingly, an extended N‐terminal linker (MGPGSGS‐UreI) was shown to restore WT *Hp*UreI functionality (Figure 3). Thus, it can be concluded that the position of the tag, as well as the length of the linker and its amino acid composition, are important factors that influence protein functionality.

In order to identify a molecular reason for the functional variation of N‐terminal constructs (HTUcl, UreIpcl), we considered the potential effect of posttranslational modifications and the charge of the N‐terminal amino group, given that both variants do not vary in the number of de/protonatable residues. During the process of protein translation, the methionine residue located at the N‐terminus of the protein is cleaved by the enzyme methionine aminopeptidase (MAP).^[^ ^65^ ^]^ In the case of HTUcl, following cotranslational cleavage, a serine and the charge of the amine group are retained at the N‐terminus. In the case of UreIpcl, the charge of the amine group is located five amino acids further into the periplasm from the aforementioned serine. The phosphorylation sites for both N‐terminal constructs were predicted using the NetPhosYeast web tool^[^ ^66^ ^]^ (Figure S8, Supporting Information), which identifies the amino acid residues that are most likely to be phosphorylated. For both constructs, the serine directly preceding the start methionine was identified as phosphorylation sites. In contrast, the remaining constructs, including the WT, lack this serine and therefore this potential phosphorylation site. Consequently, the positive charge from the N‐terminus is in close proximity to the predicted phosphorylation site in HTUcl and five amino acid residues apart in UreIpcl. The increased distance, in conjunction with linker flexibility, may potentially influence interactions within the N‐terminus and other periplasmic structural features between HTUcl and UreIpcl.

### 3.2 Ammonia Complementation Assay

**Figure 5:**
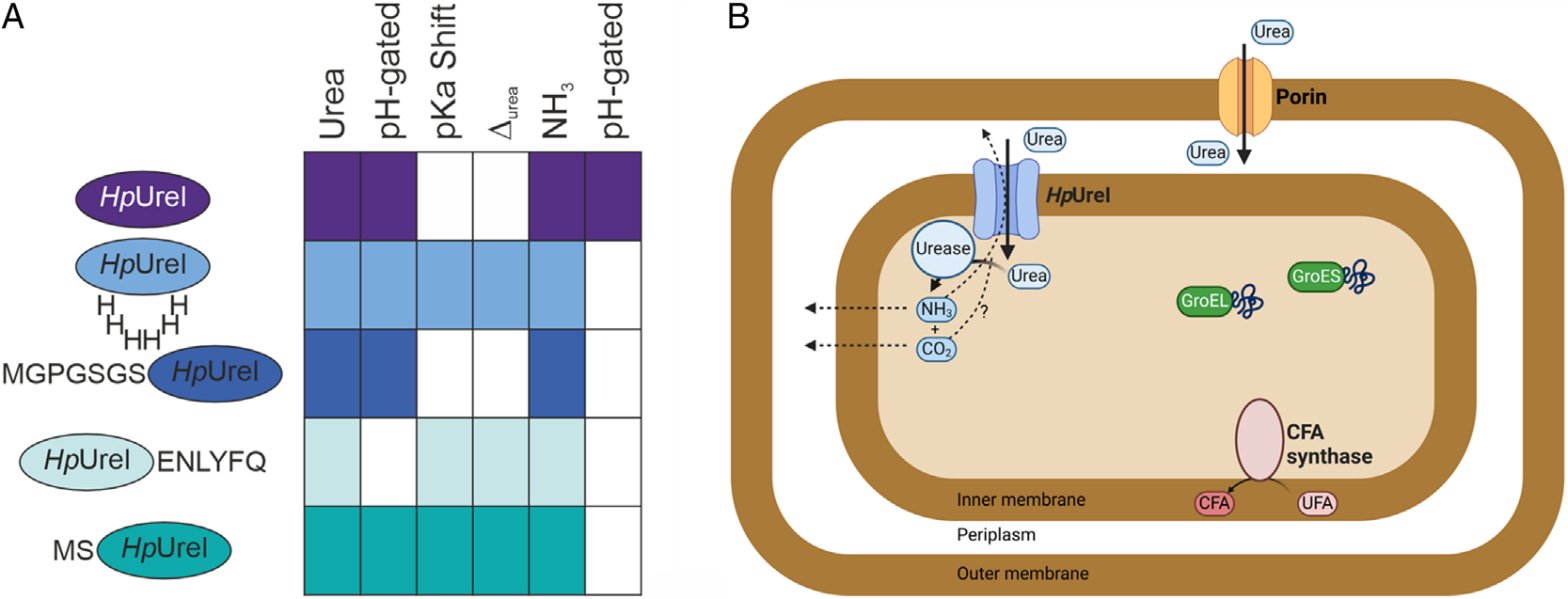
Overview of A) functional variations between *Hp*UreI tag constructs and the B) acid acclimation strategy of *Helicobacter pylori* involving several key processes. Urea gains entry into the periplasm via porins located in the outer membrane. The UreI protein plays a fundamental role in facilitating the pH‐dependent permeability of urea into the cytoplasm, where it is subsequently hydrolyzed by urease into ammonia and carbon dioxide. The resulting products serve to buffer the cytosol and periplasm. They can either passively diffuse back into the periplasm or as shown in the case of ammonia, permeate via the UreI channel (dotted lines). In addition, the bacterium utilizes heat shock proteins, GroEL and GroES, to facilitate the repair of proteins that have been damaged by acid stress. An additional adaptive strategy is the modification of the cell membrane through the action of cyclopropane fatty acid (CFA) synthase, which reduces proton permeability by converting unsaturated fatty acids into CFAs.^[^ ^70^ ^]^

Interestingly, the relative pH‐dependent ammonia permeabilities of WT *Hp*UreI and the tag variants exhibit a marked divergence from the respective urea permeabilities. While the WT shows a pH‐dependent ammonia permeability with a pKa of ≈5.7, *Hp*UreI still permits the passage of ammonia at neutral pH, albeit at a reduced rate. All tag variants demonstrate a pH‐independent intermediate ammonia permeability that is analogous to that observed for the WT protein at neutral pH. The collective findings of the urea and ammonia complementation assays **Figure** **5**A suggest that *Hp*UreI possesses a solute‐specific gating mechanism. While the protonation and deprotonation of titratable amino acid residues trigger a structural rearrangement of *Hp*UreI, the response to urea and ammonia permeation is markedly different. The slightest alteration to the *Hp*UreI WT structure results in an invariant pH‐independent ammonia permeability, whereas the effects on urea permeation are highly variable as demonstrated above.

## 4 Conclusion

In summary, our results suggest that different structural elements are crucial for the selectivity of *Hp*UreI with regard to ammonia and urea. In addition to representing a novel discovery in the field of urea channels, the observation of a solute‐specific gating mechanism may be a common feature of membrane channels. This hypothesis is supported by molecular dynamics simulations of a human aquaglyceroporin. The permeation of glycerol through *h*AQP10 was demonstrated to be pH dependent, with higher rates observed at acidic pH, whereas the permeability of water remained unchanged at low pH.^[^ ^67^ ^]^ With regard to the acid acclimation strategy of *Hp*UreI Figure 5B, our findings indicate that following the hydrolysis of urea by cytoplasmic urease, ammonia back diffusion to the periplasm is not dependent solely on passive diffusion across the inner membrane. Instead, it can directly permeate *Hp*UreI, thereby assisting in the buffering of the periplasmic pH via protonation to ammonium.

The pronounced impact of the tag position on the functionality of *Hp*UreI for urea and ammonia conductance highlights the vital importance of a screening process prior to the downstream in vitro characterization of overexpressed and purified *Hp*UreI. The impact of tag positioning on functionality has been previously demonstrated in numerous published studies. However, the evaluation of constructs for their native function remains a relatively uncommon practice. This ultimately leads to the generation of inconclusive and unreliable data for proteins, as in some instances, artificial characteristics are documented. In some studies, the protein with the tag present is tested against the cleaved version. Nevertheless, this approach may not accurately reflect the native function as illustrated by the case of *Hp*UreI. Urea permeability is altered even by the presence of a single additional amino acid at the N‐terminus. Accordingly, the untagged WT protein must be compared to the versions of the protein with a cleaved tag. This can be accomplished through the utilization of in vivo assays. As demonstrated, yeast complementation assays provide a cost‐effective, straightforward, and rapid in vivo system for the functional evaluation of multiple constructs concurrently, with adaptability to a diverse range of solutes.

## 5 Experimental Section

5.1

5.1.1

##### Yeast Strains & Plasmids

*Hp*UreI (Uniprot Accession number: P56874) constructs with differing tags were cloned into the pYES2 vector (Figure S8, Supporting Information). The residual amino acid residues after cleavage with tobacoo etch virus (TEV) protease were directly added at the C‐terminus (ENLYFQ) and the N‐terminus (S) or after cleavage with the human rhino virus (HRV) 3C protease including a short linker sequence (GPGSGS) to the N‐terminus. In a construct analogous to that used for *Hp*UreI crystallization,^[^ ^20^, ^21^ ^]^ the 6x His‐tag was incorporated into PL1 of the protein (Figure 1C). The plasmids were transformed into the yeast strain *S. cerevisiae* YNVW1, in which the endogenous urea channel DUR3 was deleted, for the urea permeability assays. To assess ammonia permeability, the endogenous ammonia transporters MEP1‐3 were deleted in the strain Sc18‐Δ*mep1‐3*.

##### Transformation

The plasmids were transformed following the instructions provided by the manufacturer of the yeast transformation kit (Sigma Aldrich) into competent cells of the specified strain. The methodology employed by the kit was based on the lithium‐acetate method.^[^ ^68^ ^]^ The competent cells were prepared by incubating them in a buffered solution of 100 mM lithium acetate. The plasmids were transformed into the cells by subjecting a mixture containing carrier DNA and PEG buffer to heat shock for 15 min at 42 °C. The positive clones were then selected on DOB‐ura plates.

##### Urea Growth Assay

For each experimental trial, a new colony was inoculated overnight (ON) in 5 mL DOB‐ura at 30 °C by shaking at 190 rpm. On the subsequent day, the cultures were diluted and cultivated until a cell concentration of 3.0 × 10^6^ cells ml^−1^ (mid‐log phase) was reached. Subsequently, the samples were centrifuged, and the resulting pellet was washed with deionized water to remove any residual glucose remaining in the media. To induce overexpression of the *Hp*UreI constructs, the samples were incubated ON in 5 mL galactose media (2% galactose, yeast nitrogen base (YNB) without ammonium sulfate and amino acids, 1 mM arginine, complete synthetic medium without uracil) at 30 °C by shaking at 190 rpm.

The overnight‐induced cells were quantified using a cell counter (cellDrop FL, Denovix) in bright‐field mode. For the seeding of cells into urea pH media, the volume was adjusted so that all constructs began with an identical cell number (between 1.4 and 5.0 × 10^6^ cells ml^−1^). The urea pH samples were subjected to centrifugation at 7400 × g for 2.5 min, after which the resulting pellet was dissolved in 5 mL of urea pH media (2% galactose, YNB without ammonium sulfate and amino acids, 100 mM succinate‐Bis‐Tris‐MOPS, 2 mM urea) with pH values of 4.0, 4.5, 5.0, 5.5, 6.0, 6.5, and 7.0 or control media arginine (2% galactose, YNB, 100 mM succinate‐Bis‐Tris‐MOPS, 1 mM arginine) at a pH of 6.0. Urea media were buffered with a mixture of buffer components consisting of sodium succinate dibasic, Bis‐Tris, and MOPS. This provided effective buffering over the entire pH range of 4.0–7.0. The pH of the media was adjusted from an initial pH of 5.7 to the required pH by the addition of HCl and NaOH. The cultures are incubated by shaking at 190 rpm at 30 °C for 48 h. The media was exchanged every 24 h for continuous cultivation. On day two after seeding, the cell concentration was measured in duplicate with the cell counter every 2.5 h, four times. An overview of the workflow of the urea growth assay is provided in Figure S9, Supporting Information. Samples (2 mL) were collected for subsequent Western blot analysis and stored at −80 °C.

##### Ammonia Growth Assay

The ammonia assay follows the same workflow as the urea growth assay (Figure S9, Supporting Information), with the exception that the cells (Δ*mep1‐3* transformants) were seeded in ammonia pH media (2% galactose, YNB without ammonium sulfate and amino acids, 100 mM succinate‐Bis‐Tris, 2 mM ammonium chloride) with pH values of 4.0, 4.5, 5.0, 5.5, 6.0, 6.5, and 7.0 or control media arginine (2% galactose, YNB, 100 mM succinate‐Bis‐Tris, 1 mM arginine) at a pH of 6.0. The ammonia pH media were buffered over the entire range of pH 4.0–7.0 with a mixture of sodium succinate dibasic and Bis‐Tris. The pH of the media was adjusted by the addition of HCl or NaOH.

##### pKa Estimation

In order to obtain the relative urea permeability, the day‐two average of each culture was normalized in relation to the arginine average cell concentration. The pH dependency plot depicts the mean of a minimum of seven independent urea pH assays. The data were fitted in OriginPro 2022 with a sigmoidal fit using the Boltzmann function.

(1) $$y=A_{2}+\frac{\left(A_{1}-A_{2}\right)}{e^{\frac{\left(x-x_{0}\right)}{dx}}}$$

*x*_0_ is the infliction point of the sigmoidal curve and thus the pKa. The fitted pKa values were presented in a bar chart. The change in relative urea permeability, Δ_urea_, between the open and the closed state of the channel is defined as

(2) $$\Delta_{\text{urea}}=A_{1}-A_{2}$$

In the case of ammonia pH assays, the data from four independent assays were averaged. The normalized cell count was calculated following the aforementioned methodology. In contrast to the presentation of urea measurements, the ammonia data were presented using spline lines with OriginPro 2022.

##### Statistical Analysis

The processing of the data was explained in the “pKa estimation” section above. No outliers were rejected in the analysis. The analysis for the independent urea and ammonia complementation assays was performed separately with sample sizes of *n* = 7 and *n* = 4, respectively. Errors were expressed as standard errors of means.

##### Western Blot Sample Preparation for Urea Complementation Assays

The pH 4.0 samples of the urea growth assay were analyzed for their protein expression level by Western blot. Sample volumes were corrected to match cell concentrations (1.4 × 10^7^ cells). The samples were centrifuged at 14 000 rpm for 2 min and pellets were dissolved in 200 μL 100 mM NaOH to lyze the cell membrane. The suspension was incubated for 10 min at room temperature (RT). After another centrifugation step the pellets were dissolved in 25 μL 3x SDS‐loading buffer (250 mM Tris‐HCl pH 6.8, 1.8% sodium dodecyl sulfate (SDS) (v/v), 45% (v/v) glycerol, bromophenol blue) with protease inhibitors (complete tablets, Roche) and incubated for 30 min at RT. A 15% SDS–polyacrylamid gel electrophoresis (PAGE) was run at 150 V for 1 h.

##### Western Blot Sample Preparation for Ammonia Complementation Assays

To analyze the protein expression level, the samples of pH 4.0 of the ammonia growth assay were adjusted to a cell density of 6 × 10^7^ cells for the Western blot. The samples were centrifuged at 14 000 rpm for 2 min. The pellets were dissolved in 1 mL ice‐cold deionized water and trichloroacetic acid (TCA)^[^ ^69^ ^]^ was added to a final concentration of 10% while vortexing. The suspension was incubated for 30 min on ice. After another centrifugation step at 14 000 rpm for 1 min at RT, the pellets were dissolved in 70 μL 2x urea buffer (6M urea, 150 mM Tris pH 6.8, 0.01% (w/v) bromophenol blue, 10% (v/v) glycerol) and about ≈50 μL of acid‐washed glass beads were added. The samples were vortexed at maximum speed for 5 min to break the yeast cells and incubated at 42 °C for 5 min. 70 μL of 2x urea buffer supplemented with 100 mM DTT was added to each sample. After vortexing at maximum speed for 5 min and incubating at 42 °C for 5 min, the samples were centrifuged at 14 000 rpm for 5 min. Supernatants were loaded on a 15% SDS–PAGE and run at 150 V for 1 h.

##### Western Blot

The SDS–gel was blotted on a nitrocellulose (Amersham) membrane in a semidry trans blotter at 25 V for 70 min. Afterward, the membrane was blocked for 1 h at RT using 5% blocking solution (skim fat dry milk powder in TBS pH 7.4), rabbit anti‐*Hp*UreI antibody (Proteogenix) was added, diluted 1:100 in 5% blocking solution, and incubated ON at 4 °C similar to Strugatsky et al. 2013.^[^ ^21^ ^]^ The membrane was washed four times with TBS pH 7.4 followed by incubation with 1: 5000 goat antirabbit IgG + HRP antibody (ThermoS) for 1 h at RT. After washing the blot four times with TBS, the chemiluminescence reaction was performed using ECL substrate mixture (Abcam). The blot was imaged in high‐resolution mode (Biorad) (Figure S4, S7, Supporting Information).

## Conflict of Interest

The authors declare no conflict of interest.

## Author Contributions

**Anna Stoib**: data curation (lead); investigation (lead); methodology (lead); validation (lead); visualization (equal); writing—original draft (equal); writing—review and editing (equal). **Sahar Shojaei**: investigation (supporting); methodology (supporting); writing—review and editing (supporting). **Christine Siligan**: investigation (supporting); writing—review and editing (supporting). **Andreas Horner**: conceptualization (lead); funding acquisition (lead); project administration (lead); supervision (lead); visualization (equal); writing—original draft (lead); writing—review and editing (equal).

## Supporting information

Supplementary Material

## Data Availability

The data that substantiate the findings of this study are available from the corresponding author upon reasonable request.
