# Supplementary material for: Yeast Complementation Assays Demonstrating the Importance of the Affinity Tag Position in Membrane Protein Purification, as Exemplified by HpUreI, the pH‐Gated Urea Channel of Helicobacter pylori
Source: Small Sci. 2025 Jan 27;5(5):2400571. doi: 10.1002/smsc.202400571 (PMC12087780; doi:10.1002/smsc.202400571)
Supplement: Supplementary file 1 — Supplementary Material [file SMSC-5-2400571-s001.pdf]

Supporting Information

Yeast Complementation Assays Demonstrating the Importance of the Affinity Tag Position in Membrane Protein Purification, as Exemplified by *Hp*UreI, the pH-Gated Urea Channel of *Helicobacter pylori.*

*Anna Stoib, Sahar Shojaei, Christine Siligan, Andreas Horner**

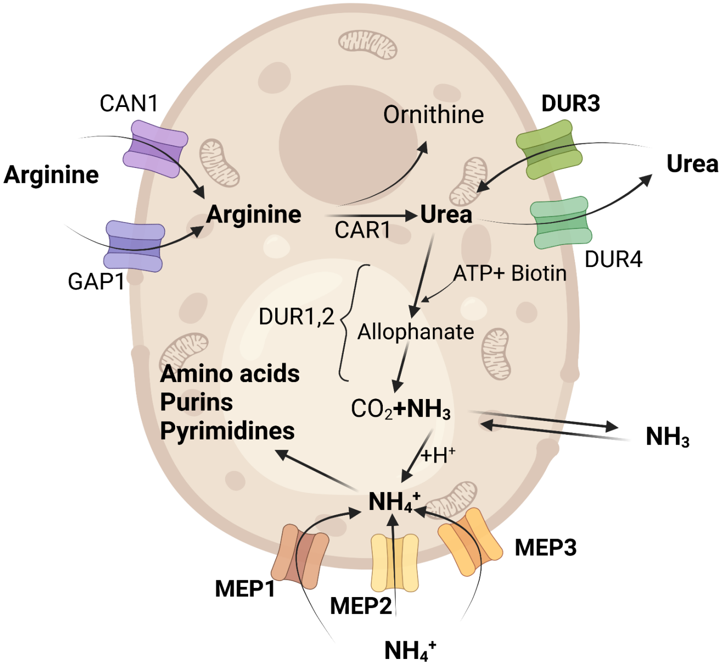

Figure S1. Scheme of arginine metabolism of *S.cerevisiae*. Arginine is taken up into the cytosol by amino acid permeases GAP1 and CAN1. Subsequently, arginine is cleaved by arginase CAR1 to urea and ornithine. Urea permeability into and out of the cell is facilitated by DUR3 and DUR4, respectively. The cell hydrolyses urea with the help of bi-functional enzyme urea amidolyases (DUR1,2) first with ATP and Biotin into the intermediate Allophanate and then into ammonia and carbon dioxide[1, 2]. Ammonia as an uncharged small molecule can diffuse through cell membranes exhibiting a low activation barrier[3]. The charged ammonium is transported by MEP1-3 into the cell[4] and is a key building block for amino acids, purines, and pyrimidines[1, 5]. In case of excessive ammonium, amino acids are secreted out of the cell[4, 5].

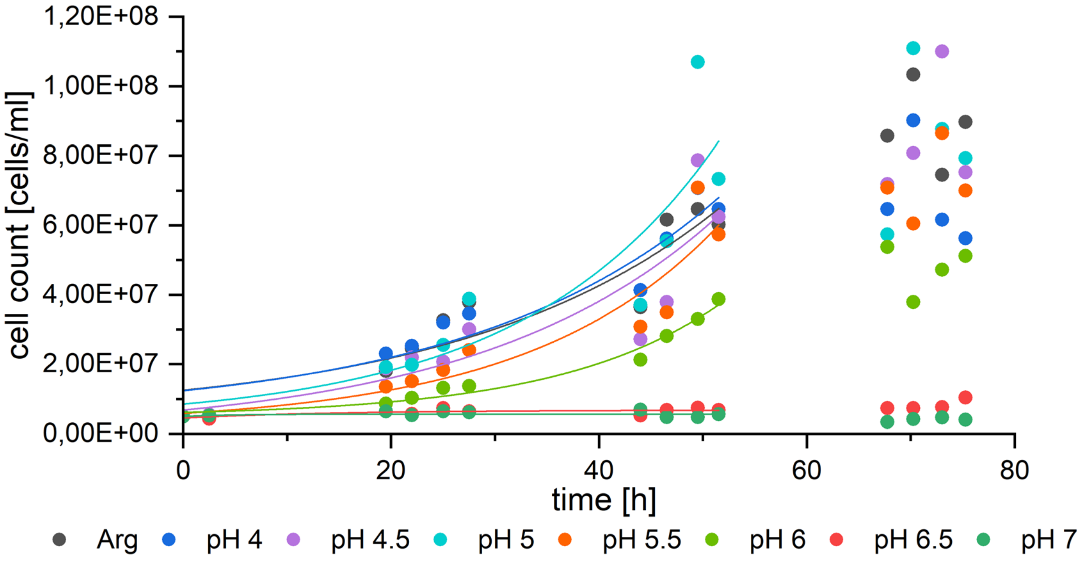

Figure S2. pH-dependent growth curves of WT *Hp*UreI utilizing urea complementation assays. Cell count is tracked for 3 days after seeding. The decline in cell count on day 3 indicates that a significant number of cells are dying, which suggests that day 2 (after approximately 48 hours) is the optimal time for measurements. As expected, the growth curves show a pH dependence, exhibiting minimal growth at a neutral pH and accelerated growth in the acidic pH range.

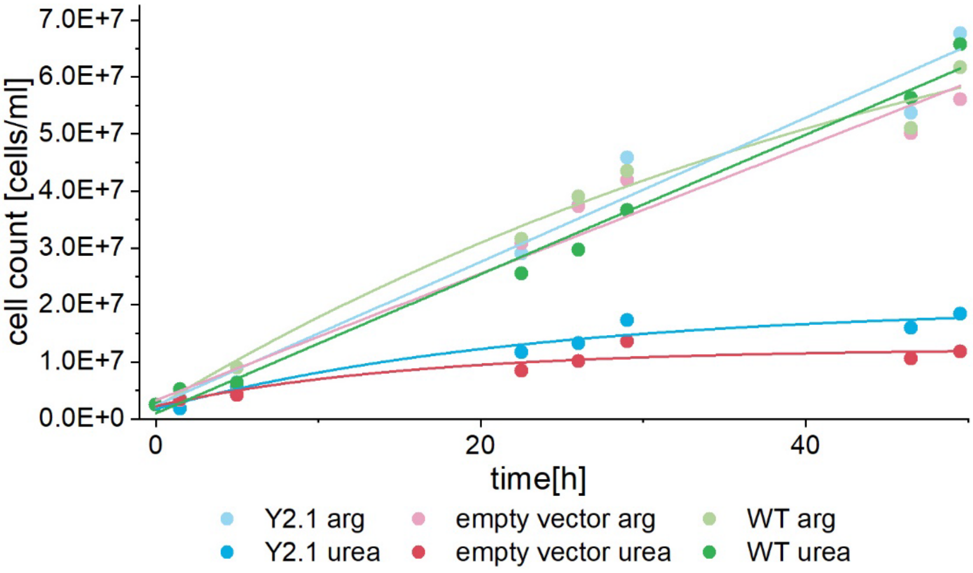

Figure S3. Control growth curves of the urea complementation assay. Growth curve of deletion strain Y2.1(*Δdur3*) without plasmid and transformed with empty vector and WT *Hp*UreI in 2mM urea and 1mM arginine media at pH 5.5. The urea-permeable channel *Hp*UreI WT exerts an influence, as the deletion strain transformed with WT *Hp*UreI is capable of growth in urea and arginine media.

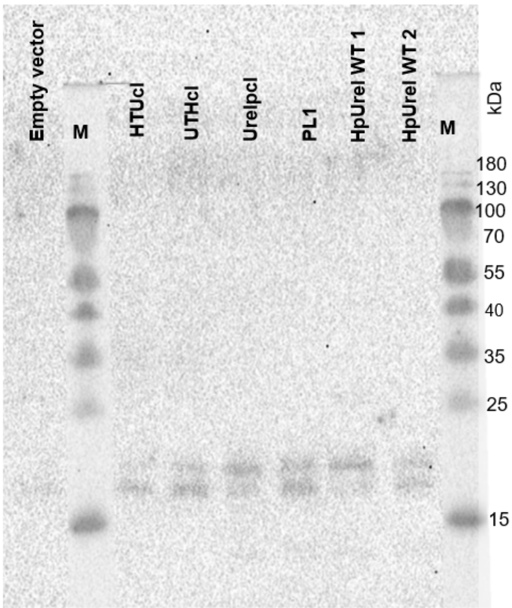

Figure S4. Exemplary Western blot with anti-*Hp*UreI of yeast cell lysates from urea complementation assays. The expression level of all constructs and the empty vector as a negative control at pH 4.0 are compared. Monomer bands are detected for all constructs at around 20 kDa corresponding to the calculated molecular weight of 21.7 kDa of a *Hp*UreI monomer[6]. The lower band is also present in the empty vector suggesting that the antibody is unspecifically binding to another yeast protein. The image of the marker lanes was merged with the chemiluminescence image. The *Hp*UreI WT 1 and 2 samples are from independent assays but the same construct, demonstrating that small variations in protein expression can occur between assays.

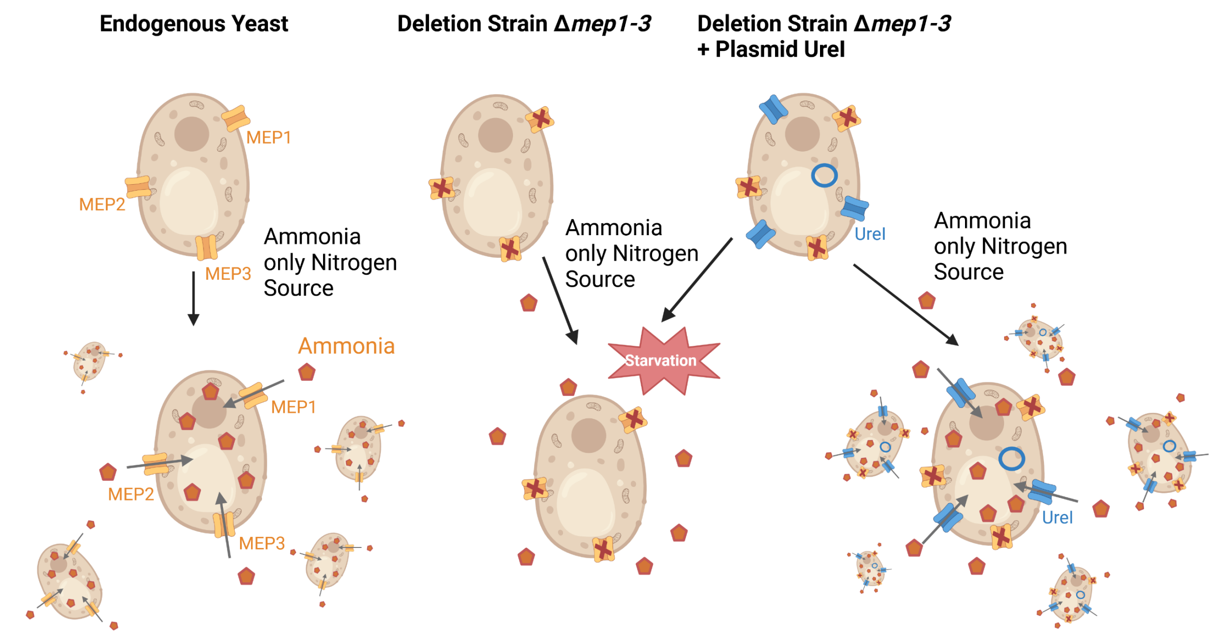

Figure S5. Principle of ammonia complementation assays in *Saccharomyces cerevisiae*. The endogenous yeast is capable of surviving in conditions where ammonia serves as the sole nitrogen source, due to the presence of transporters MEP1-3, which facilitate the permeation of ammonia into the cell. In a *Δmep1-3* deletion strain, the inability to import ammonia as a nitrogen source results in cellular starvation and a reduction in growth. Following the transformation of the deletion strain with a potential ammonia channel, the functionality, and ammonia permeability can be deduced from the observed cell growth. The greater the

permeability of the channel for ammonia, the more favorable the growth of the cells, as the channel compensates for the absence of MEP1-3[7-9].

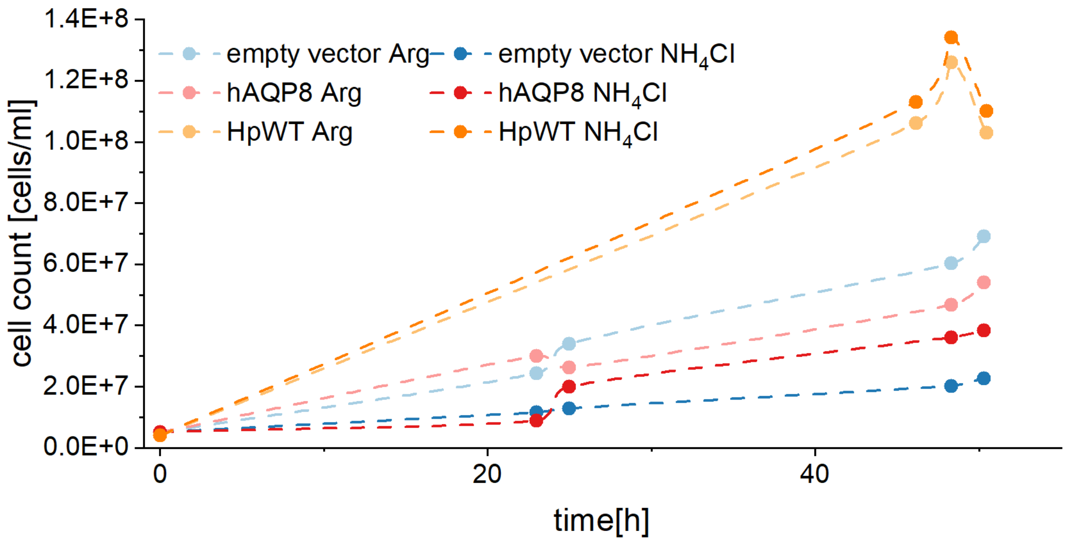

Figure S6. Control growth curves of the ammonia complementation assay. Growth curve of empty vector pYES2, *h*AQP8, and *Hp*UreI WT in 2mM Arg media (pH 6.0) and 2mM NH_4Cl^- (pH 4.5) in *Δmep1-3* yeast cells. Cell count is tracked for 2 days after seeding. It can be observed that the deletion strain is capable of growing in the ammonia and arginine media only when transformed with *h*AQP8, a human ammonia-facilitating aquaporin, or *Hp*UreI WT. This is because these channels are permeable to ammonia and can complement the deleted MEP1-3.

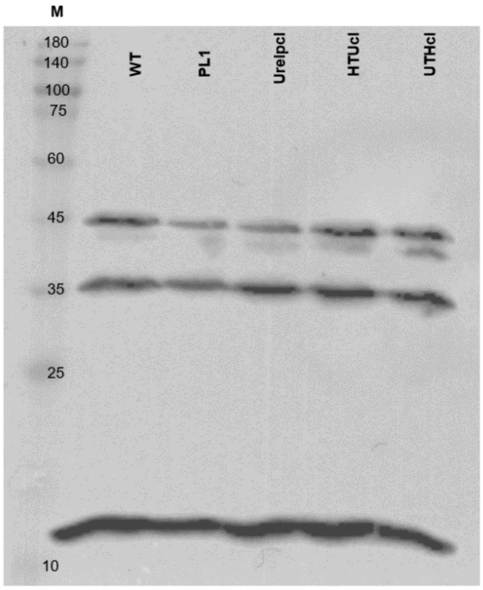

Figure S7. Exemplary Western blot with anti-*Hp*UreI of yeast cell lysates from ammonia complementation assays. The expression levels of all constructs at pH 4.0 are compared. *Hp*UreI-specific bands in different oligomeric states are detected at 35 and 45 kDa. The lower band is also present in the urea assay western blot (**Figure S3**) where it was observed as unspecific binding of the antibody. The difference to the urea samples in the oligomeric and running pattern might be caused by different loading buffer compositions.

```
PL1       -------MLGLVLLYVGIVLISNGICGLTKVDPKSTAVMNFFVGGLSIVCNVVVI 48
UTHcl     -------MLGLVLLYVGIVLISNGICGLTKVDPKSTAVMNFFVGGLSIVCNVVVI 48
HTUcl     ------MSLGLVLLYVGIVLISNGICGLTKVDPKSTAVMNFFVGGLSIVCNVVVI 49
HpUreIWT -------MLGLVLLYVGIVLISNGICGLTKVDPKSTAVMNFFVGGLSIVCNVVVI 48
UreIpcl   MGPGSGSMLGLVLLYVGIVLISNGICGLTKVDPKSTAVMNFFVGGLSIVCNVVVI 55

PL1       TYSALHPTAPVEGHHHHHHAEDIVQVSHHLTSFYGPATGLLFGFTYLYAAINHTF 103
UTHcl     TYSALHPTAPVEG------AEDIVQVSHHLTSFYGPATGLLFGFTYLYAAINHTF 97
HTUcl     TYSALHPTAPVEG------AEDIVQVSHHLTSFYGPATGLLFGFTYLYAAINHTF 98
HpUreIWT TYSALHPTAPVEG------AEDIVQVSHHLTSFYGPATGLLFGFTYLYAAINHTF 97
UreIpcl   TYSALHPTAPVEG------AEDIVQVSHHLTSFYGPATGLLFGFTYLYAAINHTF 104

PL1       GLDWRPYSWYSLFVAINTVPAAILSHYSDMLDDHKVLGITEGDWWAIIWLAWGVL 158
UTHcl     GLDWRPYSWYSLFVAINTVPAAILSHYSDMLDDHKVLGITEGDWWAIIWLAWGVL 152
HTUcl     GLDWRPYSWYSLFVAINTVPAAILSHYSDMLDDHKVLGITEGDWWAIIWLAWGVL 153
HpUreIWT GLDWRPYSWYSLFVAINTVPAAILSHYSDMLDDHKVLGITEGDWWAIIWLAWGVL 152
UreIpcl   GLDWRPYSWYSLFVAINTVPAAILSHYSDMLDDHKVLGITEGDWWAIIWLAWGVL 159

PL1       WLTAFIENILKIPKFTPWLAIIEGILTAWIPAWLLFIQHWV------ 201
UTHcl     WLTAFIENILKIPKFTPWLAIIEGILTAWIPAWLLFIQHWVENLYFQ 201
HTUcl     WLTAFIENILKIPKFTPWLAIIEGILTAWIPAWLLFIQHWV------ 196
HpUreIWT WLTAFIENILKIPKFTPWLAIIEGILTAWIPAWLLFIQHWV------ 195
UreIpcl   WLTAFIENILKIPKFTPWLAIIEGILTAWIPAWLLFIQHWV------ 202
```

Figure S8. Alignment[10] of amino acid sequences of tag constructs of *Hp*UreI. The residual tag is marked in bold. Predicted phosphorylation sites[11] are marked in red.

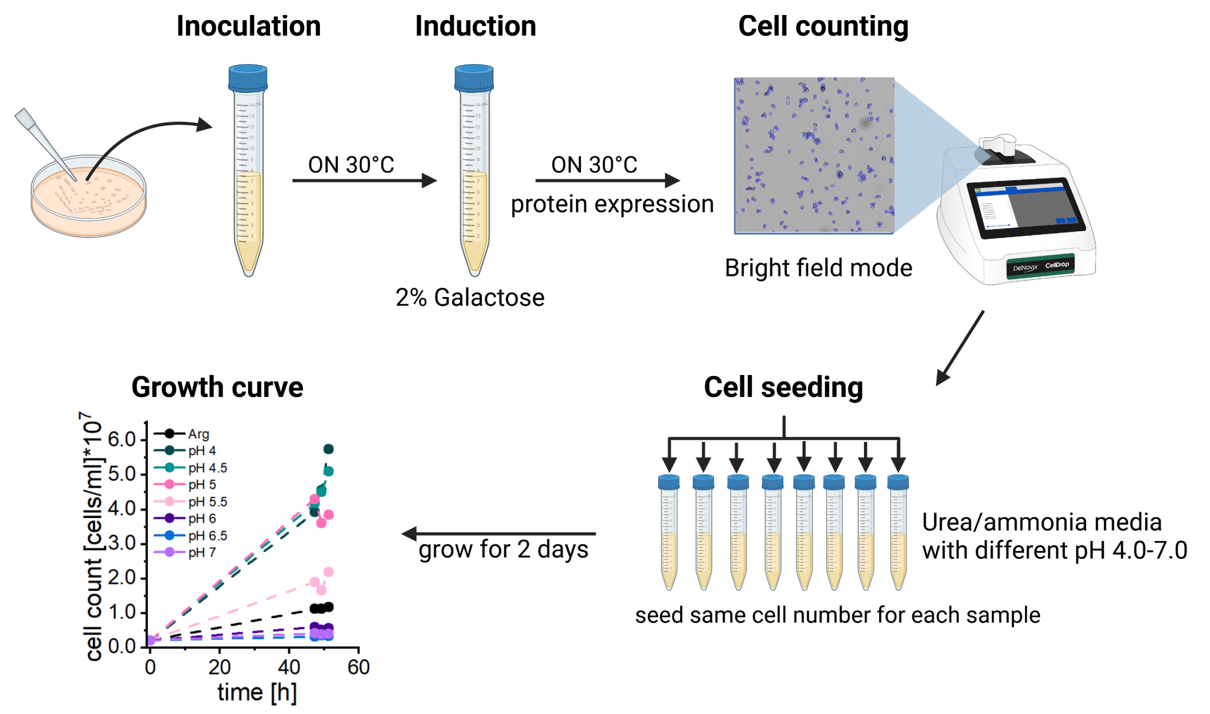

Figure S9. Detailed workflow of urea and ammonia growth assay. Single colonies of transformed constructs are inoculated, protein expression is induced by media change to galactose for 24 hours and the same cell number is seeded into urea or ammonia media with pH 4.0-7.0 and arginine media as control. Cell concentrations are measured two days after seeding.

References

[1] D. Wu, W. Xie, X. Li, G. Cai, J. Lu, and G. Xie, *Appl Microbiol Biotechnol.* **2020**, *104,* 4435-4444.

[2] D. H. Navarathna, M. S. Lionakis, M. J. Lizak, J. Munasinghe, K. W. Nickerson, and D. D. Roberts, *PLoS One.* **2012**, *7,* e48475.

[3] T. Barta, L. Seiser, and A. Horner, *Advanced Sensor Research.* **2023**, *2,*

[4] A. M. Marini, S. Vissers, A. Urrestarazu, and B. Andre, *EMBO J.* **1994**, *13,* 3456-63.

[5] P. O. Ljungdahl and B. Daignan-Fornier, *Genetics.* **2012**, *190,* 885-929.

[6] D. Strugatsky, R. McNulty, K. Munson, C. K. Chen, S. M. Soltis, G. Sachs, and H. Luecke, *Nature.* **2013**, *493,* 255-8.

[7] A. Kirscht, S. S. Kaptan, G. P. Bienert, F. Chaumont, P. Nissen, B. L. de Groot, P. Kjellbom, P. Gourdon, and U. Johanson, *PLoS Biol.* **2016**, *14,* e1002411.

[8] T. P. Jahn, A. L. Moller, T. Zeuthen, L. M. Holm, D. A. Klaerke, B. Mohsin, W. Kuhlbrandt, and J. K. Schjoerring, *FEBS Lett.* **2004**, *574,* 31-6.

[9] D. Loque, U. Ludewig, L. Yuan, and N. von Wiren, *Plant Physiol.* **2005**, *137,* 671-80.

[10] F. Sievers, A. Wilm, D. Dineen, T. J. Gibson, K. Karplus, W. Li, R. Lopez, H. McWilliam, M. Remmert, J. Soding, J. D. Thompson, and D. G. Higgins, *Mol Syst Biol.* **2011**, *7,* 539.

[11] C. R. Ingrell, M. L. Miller, O. N. Jensen, and N. Blom, *Bioinformatics.* **2007**, *23,* 895-7.
